# Functionally Significant, Rare Transcription Factor Variants in Tetralogy of Fallot

**DOI:** 10.1371/journal.pone.0095453

**Published:** 2014-08-05

**Authors:** Ana Töpf, Helen R. Griffin, Elise Glen, Rachel Soemedi, Danielle L. Brown, Darroch Hall, Thahira J. Rahman, Jyrki J. Eloranta, Christoph Jüngst, A. Graham Stuart, John O'Sullivan, Bernard D. Keavney, Judith A. Goodship

**Affiliations:** 1 Institute of Genetic Medicine, Newcastle University, Newcastle upon Tyne, United Kingdom; 2 Department of Clinical Pharmacology and Toxicology, University Hospital Zurich, Zurich, Switzerland; 3 Department of Medicine II, Saarland University Medical Center, Homburg, Germany; 4 Bristol Royal Hospital for Children, Bristol, United Kingdom; 5 Paediatric Cardiology, Freeman Hospital Newcastle upon Tyne Hospitals NHS Foundation Trust, Newcastle upon Tyne, United Kingdom; 6 Institute of Cardiovascular Sciences, The University of Manchester, Manchester, United Kingdom; New York Medical College, United States of America

## Abstract

**Objective:**

Rare variants in certain transcription factors involved in cardiac development cause Mendelian forms of congenital heart disease. The purpose of this study was to systematically assess the frequency of rare transcription factor variants in sporadic patients with the cardiac outflow tract malformation tetralogy of Fallot (TOF).

**Methods and Results:**

We sequenced the coding, 5′UTR, and 3′UTR regions of twelve transcription factor genes implicated in cardiac outflow tract development (*NKX2.5, GATA4, ISL1, TBX20, MEF2C, BOP/SMYD1, HAND2, FOXC1, FOXC2, FOXH*, *FOXA2* and *TBX1*) in 93 non-syndromic, non-Mendelian TOF cases. We also analysed Illumina Human 660W-Quad SNP Array data for copy number variants in these genes; none were detected. Four of the rare variants detected have previously been shown to affect transactivation in *in vitro* reporter assays: FOXC1 p.P297S, FOXC2 p.Q444R, FOXH1 p.S113T and TBX1 p.P43_G61del PPPPRYDPCAAAAPGAPGP. Two further rare variants, HAND2 p.A25_A26insAA and FOXC1 p.G378_G380delGGG, A488_491delAAAA, affected transactivation in *in vitro* reporter assays. Each of these six functionally significant variants was present in a single patient in the heterozygous state; each of the four for which parental samples were available were maternally inherited. Thus in the 93 TOF cases we identified six functionally significant mutations in the secondary heart field transcriptional network.

**Significance:**

This study indicates that rare genetic variants in the secondary heart field transcriptional network with functional effects on protein function occur in 3–13% of patients with TOF. This is the first report of a functionally significant HAND2 mutation in a patient with congenital heart disease.

## Introduction

Tetralogy of Fallot (TOF), the commonest cyanotic form of congenital heart disease (CHD), comprises ventricular septal defect, deviation of the outlet septum leading to over-riding of the aortic valve and right ventricular outflow tract obstruction, and right ventricular hypertrophy. Whilst approximately 20% of cases occur in the context of chromosomal abnormalities or recognised syndromes, the majority are isolated anomalies and family studies in these cases are consistent with a complex genetic model. A number of studies have tested the role of individual genes in patients with TOF; for example, rare variants in *NKX2.5* have been associated with the condition [1] but few have tested multiple genes in the same patients though this will change with the increasing application of exome sequencing [2]. When parental samples have been available an almost invariable finding has been that changes have been inherited from an unaffected parent, thus it has often been difficult to demonstrate that the variants identified contributed to the phenotype. However, transmission of alleles contributing to but not on their own sufficient to cause a phenotype is entirely consistent with the complex genetic model.

**Figure 1:**
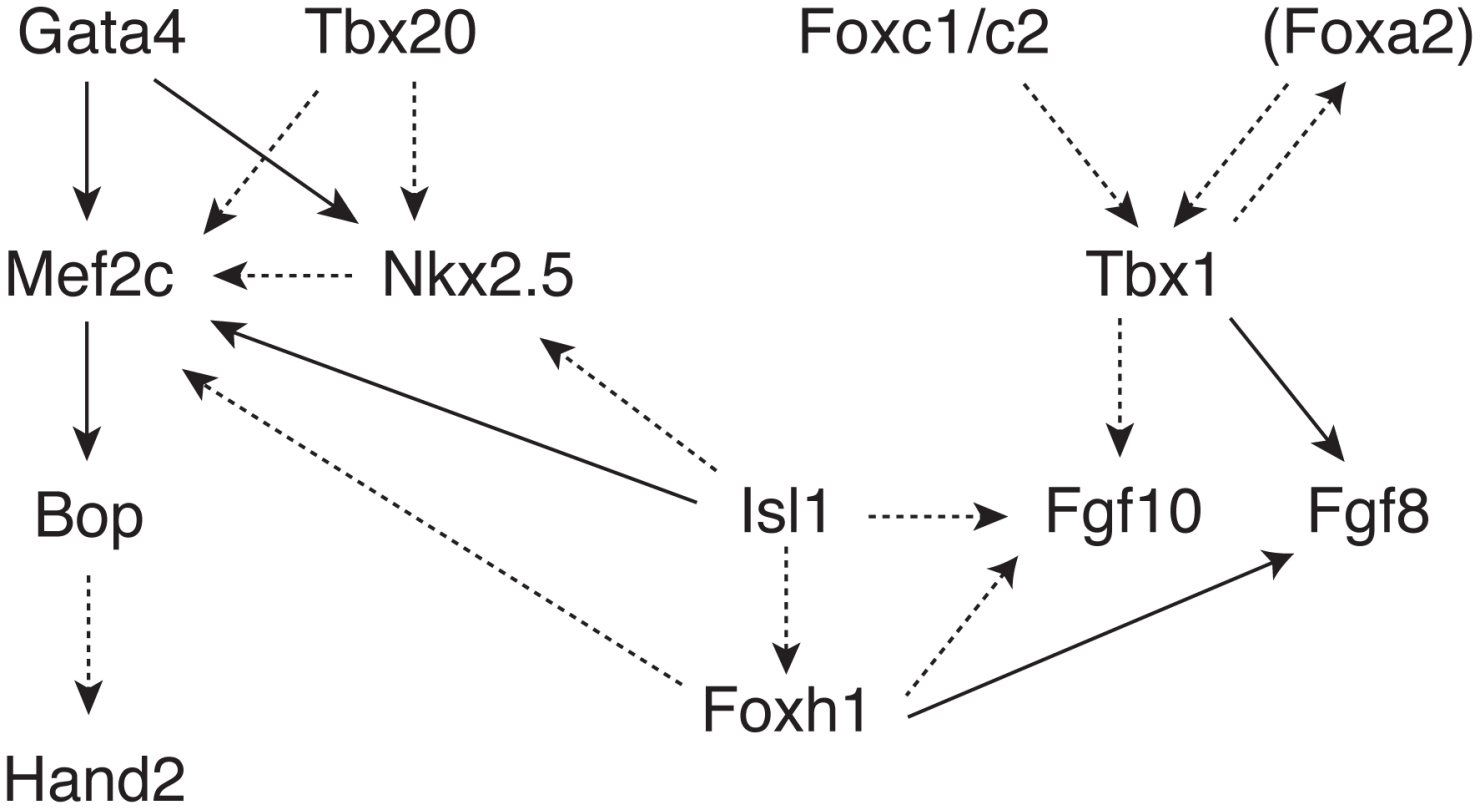
Secondary heart field transcriptional network. Solid lines indicate demonstrated direct *in vivo* activation. Dotted lines indicate genetic data or *in vitro* activation (reprinted by permission from original copyright Macmillan Publishers Ltd: Nature Genetics Reviews 6 (11): 826–835 2005). http://www.ncbi.nlm.nih.gov/pubmed/16304598.

We selected twelve transcription factors that function in the same network for study, Figure 1 [3]. Among the genes in this network, GATA4, NKX2.5 and TBX20 mutations have all been reported in familial CHD, typically septal defects [MIM 607941; MIM 108900; MIM 611363] [2], [4]–[7]. Although mutations in TBX1 have been reported [8], [9,], TBX1 hemizygosity in the context of chromosome 22q11 deletion syndrome is much more common and an important cause of CHD including TOF. Mutations in other genes in this transcription network cause disorders in which non-cardiac malformations predominate, but in which CHD may also be a feature. FOXC1 mutations cause Axenfeld Rieger syndrome type 3 (ARS) [MIM: RIEG3 602482], which is characterised by anterior chamber eye malformations and increased risk of glaucoma though CHD occurs in a significant proportion of affected individuals [10], [11]. FOXC2 mutations cause lymphoedema distachiasis syndrome [MIM 153400]; CHD is present in around ten percent of affected individuals [12]. By contrast, MEF2C hemizygosity or mutation causes severe mental retardation [13] [MIM 613443]; no patients with MEF2C mutations have been reported as having CHD. The remaining genes in the network shown in Figure 1 are not, as yet, associated with Mendelian disorders. The contribution of rare variants in these genes, considered together, in non-Mendelian, non-syndromic CHD is as yet unclear. We screened a panel of ninety three patients with TOF for mutations and assessed the functional impact of the variants we discovered. We observed rare functional and presumably deleterious variants in 6/93 patients, suggesting that such variants, while individually rare, are relatively common in TOF and may contribute importantly to disease susceptibility.

## Materials and Methods

### Ethical statement

Ethical approval was granted to the study by the NHS Multicentre Research Ethics Committee for the Northern Region (REC reference number E4/Q0902/33) and written informed consent was obtained from all participants (or their parents, if the patient was a child too young to themselves consent). We were not granted Ethics approval to undertake parental echocardiograms.

### Study population

Probands affected with Tetralogy of Fallot (TOF) were recruited from four UK cardiology centres: Leeds General Infirmary; Alder Hey Children's Hospital, Liverpool; Bristol Royal Hospital for Children and Newcastle upon Tyne NHS Hospitals Foundation Trust. Clinical records were reviewed before recruitment and probands with known chromosomal abnormalities, other recognised syndromes, learning difficulties, or known maternal exposure to significant teratogens during pregnancy were excluded. In addition, proband samples were screened for chromosome 22q11.2 deletion by multiplex ligation-dependent probe amplification (MRC-Holland, Amsterdam) to exclude DiGeorge/Velocardiofacial syndrome. All probands were of Caucasian origin and the control population consisted of ethnically-matched individuals with no history of CHD. Blood or saliva samples were collected for DNA extraction from 58 proband-parent trios, 18 proband-mother pairs, 2 proband-father pairs and 15 probands alone.

### Exon Re-sequencing

Eleven genes, namely *NKX2.5, GATA4, ISL1, TBX20, MEF2C, BOP/SMYD1, HAND2, FOXC1, FOXC2, FOXH1 and FOXA2* were re-sequenced in 93 TOF patients. *TBX1* had already been re-sequenced in the same group of patients [14]. Primers were designed based on Ensembl transcription boundaries so that amplicons would cover 5′ and 3′ untranslated, coding and splice boundary regions of all isoforms of the genes of interest and bidirectional sequencing was undertaken by standard Sanger sequencing. The Staden Package of programs (http://staden.sourceforge.net/) was used to analyse the sequence traces. Variants observed in the TOF panel that were not present in dbSNP build 131 were screened by genotyping in a cohort of 500 unrelated people free of CHD. This was carried out by the most appropriate method in each case, including iPLEX MALDI-TOF assays (Sequenom), custom Allelic Discrimination Taqman probes (Applied Biosciences), capillary electrophoresis of fluorescent-labelled amplicons, primer specific PCR and/or RFLP assays.

### Copy number variation

Ninety (of 93 screened) TOF subjects, along with 737 controls (unaffected members of TOF families), were typed on Illumina Human 660W-Quad SNP Array (Illumina Inc., San Diego, CA, USA). Genotyping was performed at Centre National de Génotypage (Evry Cedex, France). After initial QC analyses, 28 individuals were excluded due to low calls or high heterozygosity. The remaining 86 TOF individuals and 713 controls were subjected for further analyses of copy number variation as described previously [15]. Dosage analysis for *NKX2.5, GATA4, ISL1, TBX20, MEF2C, BOP/SMYD1, HAND2, FOXH1, FOXA2, FOXC1, FOXC2* and *TBX1* was carried out using Ensembl transcription boundaries of all available isoforms that were retrieved at hg18 build at UCSC Table Browser as of October 11, 2010. All co-ordinates were mapped to NCBI build 36.1 (hg18). Coverage for the genes analysed in the Illumina 660W-Quad SNP Array is given in table S1.

### Expression constructs and luciferase assays

Full-length human cDNA expression constructs HAND2-pcDNA3.1, HNF-3β-pcDNA3.1 (FOXA2), pcDNA4/HISMAXvB-FOXC1, and mouse pcDNA3.1-TBX20 have been described previously [16]–[19]. The non-synonymous rare variants found in TOF patients in the coding regions of transcription factors *HAND2, FOXA2, FOXC1*, and *TBX20* were introduced by site-directed mutagenesis (QuickChange, Stratagene) into the respective expression constructs. A luciferase reporter driven by an appropriate promoter or binding region was used in combination with each expression construct in order to measure the transcriptional activity of the expressed wild type (or mutant) transcription factors. Cells were seeded in 12-well plates for 24 hours before being transfected with empty vector, wild type or mutant expression construct along with the respective reporter construct. In addition, a GFP expression vector or Renilla luciferase vector was co-transfected into the cells as a control for transfection efficiency. Cells were incubated at 37°C and harvested 24 or 48 hours after transfection. Luciferase activity of the lysates was measured using the Dual-Luciferase Reporter Assay System (Promega) according to the manufacturer's instructions (but without addition of the Stop and Glow reagent in cases where GFP was used as control). Transfection details of each specific assay are given in table S2. GFP fluorescence was measured on the Fluoroskan Ascent FL (ThermoScientific) using excitation at 485 nm and emission at 538 nm. Luciferase data was normalised to the transfection control (GFP fluorescence or Renilla luciferase readings). For each construct, a minimum of three independent experiments were each performed in triplicate. Results are expressed as mean ± standard deviation. Statistical significance was determined by two sample t-test assuming equal variance.

### Minigene constructs and RT-PCR

The rare synonymous variants found in the *GATA4 and ISL1* genes were studied by minigene assays (exon trapping) to establish whether they impaired correct splicing. The remaining rare synonymous variants were not studied as they were located in exons not subjected to splicing. Using primers incorporating EcoRI restriction sites, each exon of interest plus 400 bp of its flanking intronic region was amplified from patient DNA and cloned into the MfeI site of construct pXJ41 [20]. pXJ41 contains a CMV promoter and two constitutive small β-globin exons. The wild type (or mutant) amplicon containing the relevant exon and its flanking intronic region was inserted between the β-globin exons. For each assay, 1 µg of the wild type (or variant) minigene construct was incubated in Optimem (Invitrogen) with 1.5 µl of Fugene HD (Roche) and transfected (in duplicate) into 70% confluent HEK293 cells seeded in 6-well plates and grown in DMEM +10% FBS for 24 hr. After 24 hours cells were harvested and RNA was extracted using Trizol, the RNA was DNAse treated to avoid amplification of the remaining transfected plasmid and then reverse transcribed using primers complementary to the beta-globin exons (forward 5′-GCTCCGGATCGATCCTGAGAACT-3′ and reverse 5′-GTAACCATTATAAGCTG-3′). The relative size of mutant and wild type amplicons were visualised under UV light after 2% agarose gel electrophoresis.

## Results

### Sequencing and copy number analysis of secondary heart field genes in TOF patients

*TBX*1 sequencing in this cohort has been reported previously [14]. Sequencing the coding, untranslated and exon boundary regions of the remaining 11 transcription factor genes in 93 TOF patients identified 40 variants that were not present in dbSNP build 131. Six variants not present in dbSNP build 131 were present in the control population with a frequency of >1% (table S3).The frequency of the remaining 34 changes in the Exome Variant Server, NHLBI GO Exome Sequencing Project (ESP), Seattle, WA (URL: http://evs.gs.washington.edu/EVS/) [August 2013], 1000 Genomes (http://browser.1000genomes.org) and in our own controls are shown in Table 1. Half (17/34) of the rare variants were in coding regions, 14 were in 3′ and 5′ untranslated regions and 3 were intronic. Of the 17 coding region variants, 3 were synonymous changes and 14 were non-synonymous changes; 10 resulting in amino acid substitutions and 4 resulting in in-frame insertions or deletions. No copy number variants removing a gene or part of a gene were detected.

**Table 1:** Rare variants found in the 93 Tetralogy of Fallot patients.

Gene	Location	Base	aa change	Id	Inh.	500 controls (%)	NHLBI (%)	1000 g (%)	dbSNP 137
***FOXA2***	**intron 1**	c.-2+4_+17del	n/a	58	—	—	—	—	—
	**exon 3**	c.280G>C	p.A94P	28	M	—	—	—	rs201350646
		c.565A>T	p.I189F	40	P	—	—	—	—
		c.872C>T	p.A291V	73	P	0.2	—	—	rs200459003
		c.1334A>G	p.Y445C	35	—	—	—	—	—
		c.*471dupA	n/a (3'UTR)	71	M	—	—	—	—
***FOXC1***	**exon 1**	c.81_89del GCGGCGGCC	p.A28_A30delAAA	313	M	0.3	—	—	—
		c.889C>T	p.P297S	66	M	—	—	1.7	rs79691946
		c.1132_1140del	p.G378_G380delGGG	22	M	—	—	—	—
		c.1462_1473del	p.A488_491delAAAA	22	M	—	—	—	—
***FOXC2***	**exon 1**	c.583C>G	p.P195A	78	P	0.2	—	—	rs200751941
		c.1331A>G	p.Q444R	34	—	0.2	0.14	—	rs147258453
***FOXH1***	**5'UTR**	c.-543C>T	n/a	60	M	0.4	—		—
		c.-333T>C	n/a	76	P	0.6	—	—	rs147276162
				342	—				
		c.-136delC	n/a	69	P	0.2	—	—	—
		c.-76_88del TCAGGTCCCGGCC	n/a	29	M	—	—	—	—
	**exon 3**	c.338G>C	p.S113T	500	—	0.8	0.66	0.2	rs144830740
***GATA4***	**exon 2**	c.699G>A	p.T233T	323	—	—	0.3	0.1	rs55788387
	**intron 3-4**	c.909+25G>A	n/a	507	—	0.9	0.72	0.003	rs147860174
				509	—				
				331	M				
	**exon 5**	c.1037C>T	p.A346V	59	—	0.4	0.31	0.13	rs115372595
				61	M				
	**exon 6**	c.1164G>A	p.A388A	315	M	—	0.03	—-	rs55968178
		c.*886G>A	n/a (3'UTR)	319	M	—	—	0.4	rs146304341
		c.*979G>C	n/a (3'UTR)	73	M	0.1	—	0.13	rs182365313
		c.*1012G>C	n/a (3'UTR)	67	—	0.1	—	0.13	rs139566390
				74	—				
***HAND2***	**exon 1**	c.75_76insGCCGCC	p.A25_A26insAA	28	M	—	—	—	—
***ISL1***	**intron 2-3**	c.219-3C>T	n/a	514	M	—	—	—	—
	**3'UTR**	c.*245A>G	n/a	44	M	—	—	—	—
		c.*651A>G	n/a	39	M	0.5	—	—	—
***BOP/SMYD***	**3'UTR**	c.*140T>C	n/a	30	P	—	—	—	—
		c.*271C>T	n/a	330	P	0.1	—	—	—
		c.*699C>T	n/a	32	M	0.9	—	0.5	rs150572228
		c.*2435C>T	n/a	28	P	—	—	—	rs142969860
***TBX20***	**exon 1**	c.81C>T	p.G27G	325	M	—	0.01	—	rs113390069
	**exon 2**	c.364A>G	p.I122V	31	M	0.2	—	—	—
***TBX1***	**exon 3**	c.115G>A	p.G39S	31	M	—	—	—	—
		c.129_185del57	p.P43_G61del PPPPRYDPCAAAAPGAPGP	330	M	—	—	—	—
	**exon 9a**	c.*11C>T	n/a (3'UTR)	57	M	0.1	0.16	—	rs72646973
				73	M				
	**exon 9c**	c.*1074G>A	n/a (3'UTR)	319	M	—	—	—	—

Inh  =  Inheritance: P = paternal, M = maternal. Frequency data is shown for the 500 ethnically matched controls studied and also NHLBI (Exome Sequencing Project) and 1000genomes data (European populations only). Data for the previously reported *TBX1* variants is included.

Four non-synonymous variants were found in *FOXC1* and *FOXA2*, two in *FOXC2*, and single non-synonymous variants were found in *GATA4, TBX20, HAND2* and *FOXH1*. Each variant was present in a single patient apart from *GATA4* c.1037C>T (p.A346V) and c.*1012G>C; and *FOXH1* c.-333T>C that were found in two patients each, and the intronic *GATA4* c.909+25G>A that was found in 3 patients. We included the *TBX1* re-sequencing information for these patients [14] when considering the hypothesis that changes in more than one gene in the pathway may predispose to TOF. Most patients presented a single variant, however two variants were found in three patients. The first patient carried both *GATA4* c.*886G>A and *TBX1* c.*1074G>A (both maternal in origin); the second carried *BOP/SMYD1* c.*271C>T and *TBX1* c.129_185del, p.P43_G61del PPPPRYDPCAAAAPGAPGP (inherited from the father and mother, respectively) and the third carried both *FOXC1* c.1132_1140del, p.G378_G380delGGG and c.1462_1473del, p.A488_491delAAAA (both on the maternally derived allele). Three variants were found in two patients. The first carried *BOP/SMYD1* c.*2435C>T (paternal), *HAND2* c.75_76insGCCGCC, p.A25_A26insAA (maternal) and *FOXA2* c.280G>C, p.A94P (maternal). The second patient carried *GATA4* c.*979G>C (maternal) *FOXA2* c.872C>T, p.A291V (paternal) and *TBX1* c.*11C>T (maternal). All variants were heterozygous. Of the 28/34 rare variants for which parent of origin could be determined 20 were inherited from the mother and 8 from the father; parental samples were not available to test whether the remaining six rare variants were inherited or had occurred *de novo*. No novel changes were detected in *NKX2.5 or MEF2C*.

### Functional investigation

Four of the rare variants detected have previously been shown to affect transactivation in *in vitro* reporter assays: FOXC1 p.P297S, FOXC2 p.Q444R, FOXH1 p.S113T and TBX1 p.P43_G61del PPPPRYDPCAAAAPGAPGP. Six variants (HAND2 p.A25_A26insAA, FOXA2 p.A94P, p.I189F and p.Y445C, and FOXC1 p.G378_G380delGGG and p.A488_491delAAA) were not present in 1000 control chromosomes, NHLBI Exome Sequencing Project or 1000 Genomes. For the missense changes, *in silico* tools predicted FOXA2 p.A94P, p.I189F and p.Y445C to be damaging (with PolyPhen scores of 0.918, 1 and 1 respectively). Based on this, the transcriptional activities of HAND2 p.A25_A26insAA, FOXA2 p.A94P, p.I189F and p.Y445C, and the compound FOXC1 p.G378_G380delGGG and p.A488_491delAAAA were compared to the activities of their respective wild type transcription factors. In addition, we studied the transcriptional activity of TBX20 p.I122V as variation of the neighbouring amino acid results in gain of function [7] although it was predicted by Polyphen to be benign (0.352).

#### HAND2

**Figure 2:**
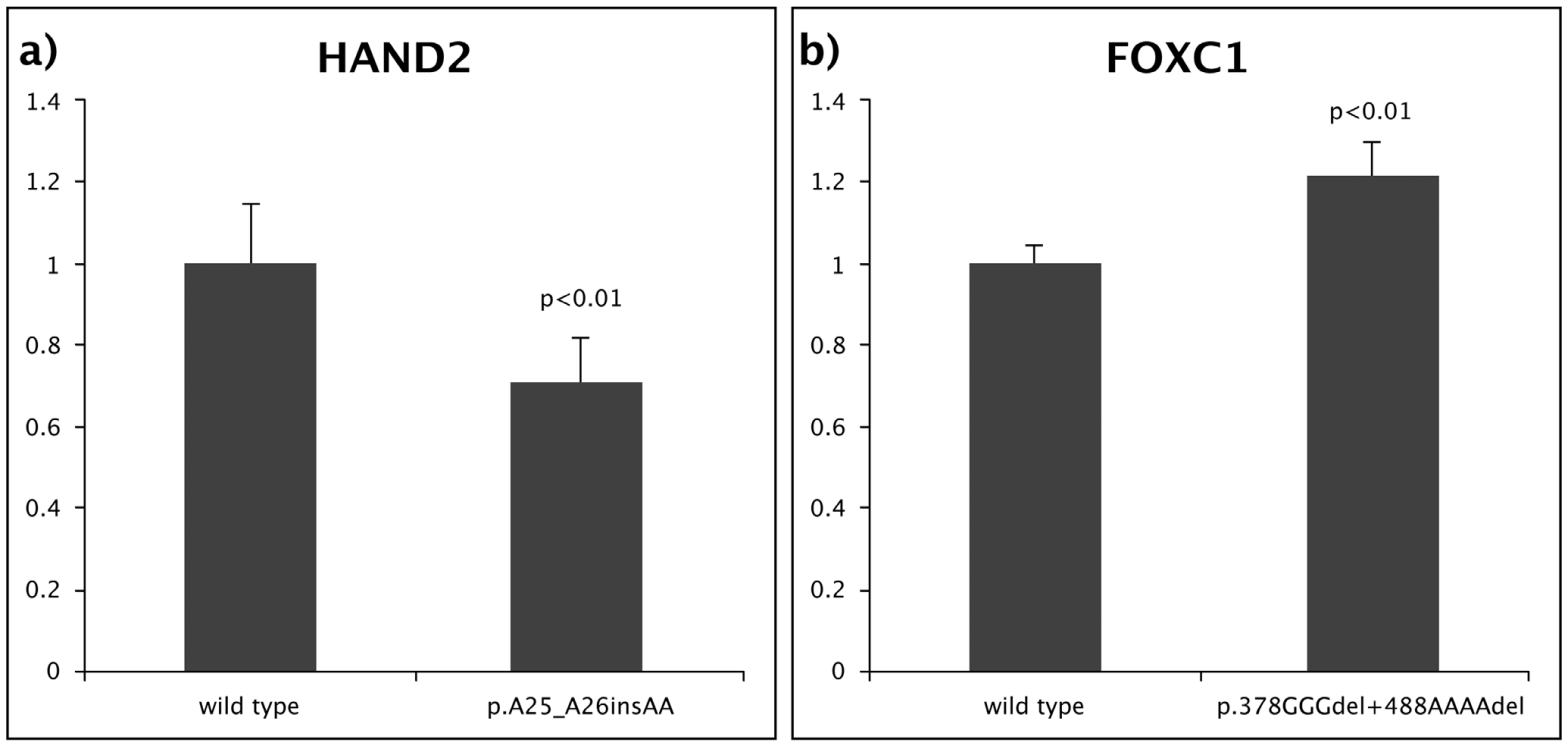
Transcriptional activity of wild type and rare non-synonymous variants of transcription factors HAND2 and FOXC1. a) The HAND2 p.A25_A26insAA variant showed significantly reduced activation of the ANP reporter construct; b) The FOXC1 p.378GGGdel+488AAAAdel variant showed significantly increased activation of the 6xBS reporter construct. All assays were repeated at least three times, each time in triplicate and standard deviations are shown.

Transient expression of HAND2 in HEK293 cells results in up-regulation of the ANP-luciferase reporter [16]. Insertion of two alanines in a tract of 12 alanines (p.A25_A26insAA) reduced the up-regulation to 80% of the wild type levels (p = 0.02, Figure 2, panel a).

#### FOXA2

Transient expression of FOXA2 in HUH7 cells results in down-regulation of the OATP8-luciferase reporter [17]. None of the three non-synonymous FOXA2 variants investigated (p.A94P, p.I189F, and p.Y445C) affected transcriptional activity as measured by this luciferase reporter assay (data not shown).

#### TBX20

Transient expression of TBX20 and constitutively active ALK3 in NIH3T3 cells results in down-regulation of a Tbx2-Luc reporter [19]. There was no difference in the down-regulation observed between wild-type TBX20 and the p.I122V variant (data not shown).

#### FOXC1

Transient expression of FOXC1 in COS7 cells results in up-regulation of the 6xBS-luciferase reporter [18]. Deletion of either three glycines or four alanines independently had no effect on FOXC1 transcriptional activity (data not shown). As the GGGdel and the AAAAdel occurred on the same allele an expression construct carrying both variants was also tested. This variant increased transcriptional activity compared to the wild type protein (p = 0.003). (Figure 2, panel b).

### Investigation of splicing

The mutant minigene constructs for *ISL1* c.219-3C>T and *GATA4* c.699G>A p.T233T produced an RNA profile identical to the wild type construct indicating that splicing was not affected by these genetic changes (data not shown).

## Discussion

Nineteen coding region variants that occurred in less than 1% of control chromosomes were identified in 93 TOF cases in the secondary heart field transcription network genes, eleven transcription factors studied here and *TBX1* analysed in the same cases previously [14]. These variants comprised eleven missense, one in-frame insertion, four in-frame deletions (one complex) and three synonymous variants; no truncating variants were found. All bar one, GATA4 p.A346V, was found in a single patient and each patient had only one of these variants. In all cases for which parental samples were available the rare variants had been inherited.

The functional effect of the novel variants that were absent from the controls was assessed by reporter assays. In the assay systems we utilised, the HAND2 insertion and the FOXC1 compound deletion were functionally significant. Four further rare variants, each present in one of the TOF cases, had been observed and studied previously and found to alter transactivation capacity: FOXC1 p.P297S, FOXC2 p.Q444R, FOXH1 p.S113T and TBX1 p.P43_G61del PPPPRYDPCAAAAPGAPGP [21]–[23] [14]. Finally, TBX20 p.I122V was also studied as a change in the adjacent amino acid (TBX20 p.I121M), identified in a patient with an ostium secundum atrial septal defect, increased transactivation [7].

We found that a variant present in one patient resulting in the insertion of two alanines in a tract of 12 alanines (p.A25_A26insAA) in HAND2 decreased transcriptional activity. Expansions of polyalanine tracts are a well-recognised cause of disease [24]. In the majority of these disorders the polyalanine tracts occur in genes encoding transcription factors. The length of the polyalanine tract is generally in the order of 10A–20A and typical expansions range from five to fourteen alanines but expansions by two alanine residues have been reported to be sufficient to cause disease [25], [26]. In addition to tetralogy of Fallot this patient had an absent thyroid gland (Table 2). A deletion in the orthologous zebrafish gene causes thyroid agenesis and an insertion in the zebrafish gene causes reduction in the size of the thyroid gland. The similarity of this additional phenotype between the zebrafish model and the patient provide further support for the pathogenicity of this HAND2 mutation [27]. There have been no previous reports of *HAND2* mutations associated with other human phenotypes and there is only one report on *HAND2* analysis in patients with CHD [28]. This study detected one missense mutation (p.P11R) in two TOF patients that was not present in 250 ethnically matched controls, no functional data was presented. A number of patients have been reported with duplications and deletions of chromosome 4q33, the chromosomal region containing *HAND2*. Patients with both duplications and deletions have a high incidence of congenital heart defects including tetralogy of Fallot consistent with dosage sensitivity for this gene in human cardiogenesis but all duplications and deletions have affected multiple genes [29], [30].

**Table 2:** Clinical information of TOF patients with rare, functionally significant variants.

Patient Id	Gene	Change	Phenotype
22	*FOXC1*	p.378GGGdel, p. 488AAAAdel	Tetralogy of Fallot with bicuspid pulmonary valve, subvalvar and valvar stenosis, hypoplasia of left pulmonary artery at insertion of ductus arteriosus, confluent branch pulmonary arteries, left aortic arch.
28	*HAND2*	p.A25_A26insAA	Tetralogy of Fallot with pulmonary atresia and major aortopulmonary collateral arteries, right aortic arch, small atrial septal defect, small central pulmonary arteries, absent thyroid gland.
34	*FOXC2*	p.Q444R	Tetralogy of Fallot with moderate valvar pulmonary stenosis and mild subvalvar pulmonary stenosis, confluent branch pulmonary arteries, pyloric stenosis.
66	*FOXC1*	p.P297S	Tetralogy of Fallot with moderate to severe subvalvar pulmonary stenosis, confluent branch pulmonary arteries, right aortic arch, patent foramen ovale.
330	*TBX1*	p.P43_G61del PPPPRYDPCAAAAPGAPGP	Tetralogy of Fallot with a dysplastic pulmonary valve, patent foramen ovale and right aortic arch, severe valvar and supravalvar pulmonary stenosis, confluent branch pulmonary arteries.
500	*FOXH1*	p.S113T	Tetralogy of Fallot with severe valvar and subvalvar pulmonary stenosis, right aortic arch, confluent branch pulmonary arteries, ligamentum arteriosum.

Three cases in the group had a *FOXC1* coding change, these were a deletion of three alanine residues that was also present in three of the controls, a missense change, *FOXC1* p.P297S, and non-contiguous deletion of three glycines and four alanine residues. None of these changes were in the forkhead domain. *FOXC1* has been intensively studied in Axenfeld Rieger syndrome (ARS) patients. FOXC1 p.P297S has been reported in two patients with this condition and reported to decrease transcriptional activity to 75% of normal levels [21]. Neither the affected child in this study nor the parent from whom the variant was inherited had a known eye problem. Furthermore this change has now been reported in the 1000 genome project suggesting that if it is a cause of ARS it is not fully penetrant. The majority of FOXC1 mutations in ARS localise to the forkhead domain and decrease transcriptional activity [31]. In contrast combined deletion of the three glycines and 4 alanines increased transcriptional activity in the reporter assay suggesting that increased activity of FOXC1 may play a role in CHD aetiology. Importantly, however, inheritance from an unaffected parent emphasises the likely requirement for additional genetic and/or environmental factors for CHD to occur. It is also noteworthy that functional testing showed no effect of each variant comprising the maternal haplotype individually, but a significant increase in transcriptional activity when a construct incorporating both variants was used. This illustrates the potential importance of incorporating multiple variants on a haplotype for functional testing, where these are encountered.

The purpose of this study was to assess the contribution of rare variants in the secondary heart field transcriptional network to TOF. We identified novel changes in HAND2 and FOXC1 that affected reporter transactivation and found changes in FOXH1, FOXC1 and FOXC2 that had previously been shown by others to have affect transactivation capacity. Interestingly these latter changes did not meet the criteria we had selected for functional analysis as they were present in controls. We had previously studied TBX1 in the same 93 patients and detected a functionally significant in-frame deletion [14]. Had our criteria been less stringent functional assays would have been undertaken for an additional three changes in GATA4, FOXC1 and FOXC2 respectively. Although the three FOXA2 changes were predicted to be damaging by PolyPhen no change was observed in the readout of the functional assays, nor for TBX20 p.I122V. More detailed phenotypic information for the six patients with functionally significant changes is shown in Table 2. It is noteworthy that four of the six patients had a right sided aortic arch, a feature seen in approximately a quarter of TOF patients [32].

Our study has shown that rare coding variants in genes in a network playing a central role in the development of right-sided cardiac structures and the outflow tract occur relatively frequently in sporadic, non-syndromic tetralogy of Fallot; the 95% confidence interval of the proportion we observed (6 functionally significant variants/93 cases) is between 3 and 13%. This suggests that a network-wise comparison of variant burden, albeit in much larger numbers of patients and controls, could demonstrate a statistically significant excess of variants among the TOF patients. Difficulties with such an approach would include the precise *a priori* definition of the gene network, and our observation that among the rare variants we detected, not all exhibited functional significance in the assays we adopted. In this regard, it is possible that the *in vitro* assays we chose do not faithfully reflect the effect of the investigated variants on embryonic development; moreover, although the functional differences we observed were reproducible and statistically significant, they were of relatively small magnitude. We did not detect examples where functional assays supported di- or polygenic inheritance, though further study in larger patient groups is needed to obtain adequate power to address combinations of changes.

## Supporting Information

Table S1 **Coverage for the genes analysed in the Illumina 660W-Quad SNP Array.**
(XLSX)

Table S2 **Transfection details of luciferase assays.**
(XLSX)

Table S3 **Variants found with a frequency of >1% in the control population.**
(XLSX)
